# Exploiting Publication Contents and Collaboration Networks for Collaborator Recommendation

**DOI:** 10.1371/journal.pone.0148492

**Published:** 2016-02-05

**Authors:** Xiangjie Kong, Huizhen Jiang, Zhuo Yang, Zhenzhen Xu, Feng Xia, Amr Tolba

**Affiliations:** 1 School of Software, Dalian University of Technology, Dalian, China; 2 Riyadh Community College, King Saud University, Riyadh, Saudi Arabia; 3 Mathematics and Computer Science Department, Faculty of Science, Menoufia University, Shebeen El-Kom, Menoufia, Egypt; Universitat Rovira i Virgili, SPAIN

## Abstract

Thanks to the proliferation of online social networks, it has become conventional for researchers to communicate and collaborate with each other. Meanwhile, one critical challenge arises, that is, how to find the most relevant and potential collaborators for each researcher? In this work, we propose a novel collaborator recommendation model called CCRec, which combines the information on researchers’ publications and collaboration network to generate better recommendation. In order to effectively identify the most potential collaborators for researchers, we adopt a topic clustering model to identify the academic domains, as well as a random walk model to compute researchers’ feature vectors. Using DBLP datasets, we conduct benchmarking experiments to examine the performance of CCRec. The experimental results show that CCRec outperforms other state-of-the-art methods in terms of precision, recall and F1 score.

## Introduction

The current scale of the Internet has risen beyond the imagination of people due to its rapid development. Consequently, how to obtain useful and effective information has become a complex task as a result of information overload. Recommender systems and techniques reduce the problems and immensely help people by providing easier access to the relevant resources they really need.

Collaboration among researchers often occurs and it has been shown that research collaboration has impact on scientific productivity [1]. Therefore, collaboration recommendation becomes very necessary and has been attracting more and more researchers in recent years. Generally, collaboration recommendation can be grouped into two classes: 1) to recommend the most potential collaborators (MPCs) who have never collaborated with the target (i.e. to build new collaborations); 2) to recommend the most valuable collaborators (MVCs) who have ever collaborated with the target before (i.e. to reinforce old collaborations). Lopes et al. [2] worked on identifying new partners to execute joint research and enhancing the collaboration of current partners for researchers. Chen et al. [3] proposed that the purpose of friends recommendation is to make new friends and keep the old ones. Research on enterprise social networking [4] shows that users in a corporate context are interested in discovering valuable contacts not yet known to them, or connecting to weak ties, in addition to staying in touch with their close colleagues. Our previous work [5, 6] focuses on recommending MVCs for researchers and enhancing the collaboration with colleagues in their academic social networks, which enables the researchers to collaborate with each other again. However, many scientists also initiate collaborations outside of their social networks. It is burdensome and fraught with risk of initiating collaboration with socially unconnected researchers. Therefore, unconnected researchers (MPCs) might be more valuable to be recommended. In this work, in contrast, CCRec has an aptitude for discovering new collaborators with high similarity (i.e. MPCs recommendation).

Considering the inherent requirements, a variety of methods related to collaborators recommendation have been proposed, which involve three main aspects: content-based, CF (collaborative filtering) based, social network-based and hybrid recommendation. Some traditional content-based methods extract researchers’ academic features through tags of interests, user profiles, publications, etc. Gollapalli et al. [7] proposed models for computing the similarity between researchers based on expertise profiles extracted from their publications and academic home pages. Lopes et al. [2] considered researchers’ publications area and the vector space model to make collaboration recommendation. CF based methods is famous and popular in recommender area. It is also well used in collaborators recommendation. Kim et al. [8] proposed a collaborative filtering method to provide an enhanced recommendation quality derived from user-created tags. Moreover, a large amount of recommender systems benefited from introducing social network. Ma et al. [9] analyzed how social network information can benefit recommender systems and proposed a method of improving the performance of recommender systems by incorporating social network information. Huynh et al. [10] proposed a method based on a combination of probability theory and graph theory for modeling and analysing co-author networks. They explored similar vertices of potential candidates for collaboration recommendation. Their main contribution involves taking the trend information into considering when computing the similarity of vertices. Many other approaches [11, 12] have been applied to large social networks to solve the link prediction problem, which is similar with relationship recommendation. Their works on social networks show good performance. Lichtenwalter et al. [13] examined some important factors for link prediction and proposed a general framework. In our previous work [5, 6], we also developed several features in academic social networks, which well enhanced the performance of our recommender model. Some hybrid collaboration recommendation models have been introduced in recent years. Lee et al. [14] exploited how well content-based, social network-based and hybrid recommendation algorithms predict co-author relationship. The given results show that a hybrid algorithm combining content and social networks information performs better. Some other brilliant hybrid algorithms can be found in [15], [16] and [17]. Recently, Chaiwanarom et al. [18] proposed a new hybrid algorithm for recommending appropriate collaborators in interdisciplinary computer science using degrees of collaborative forces, temporal evolution of research interest, and comparative seniority status. The result shows that it is effective and innovative.

Considering the real academic collaboration scene, researchers often behave differently across multiple domains of interests, which might introduce topic drift problems in general recommendation systems. Considering this problem, Tang et al. [19] focused on modeling topics with high probability of having cross domain collaboration when studying cross-domain collaborator recommendation (another hot area of academic recommendation). Furthermore, a researcher often shows bias on various academic domains. Such behaviors usually reveal academic features of researchers in different domains. Thus, it is imperative to consider academic domains when recommending collaborators. Chen et al. [20] discussed CollabSeer, an open system to recommend potential research collaborators for researchers and scientists. They discovered collaborators based on the structure of co-author networks and the user’s topic of research interests. This previous work, along with e.g. [21], stimulated our inspiration to introduce a topic model in CCRec.

Here we propose a novel hybrid model by exploiting publication contents and collaboration networks for collaborators recommendation (CCRec). In summary, we make the following contributions in this paper: 1) To compute the most potential collaborators recommendation, we develop a model CCRec, which combines the content-based and social network-based methods. By adopting this procedure, our approach is more favourable in terms of achieving remarkable personalized collaborators recommendation. 2) To reveal researchers’ academic features in different domains, we present the feature vectors by utilizing a topic clustering model and use a random walk model to compute researchers’ influence in each domain. The results show that the method is effective. 3) We conduct extensive experiments on a subset of DBLP data set to evaluate the performance of CCRec in various scenarios. Moreover, we measure four models for comparison. Promising results are presented and analyzed.

## Methods

**Fig 1:**
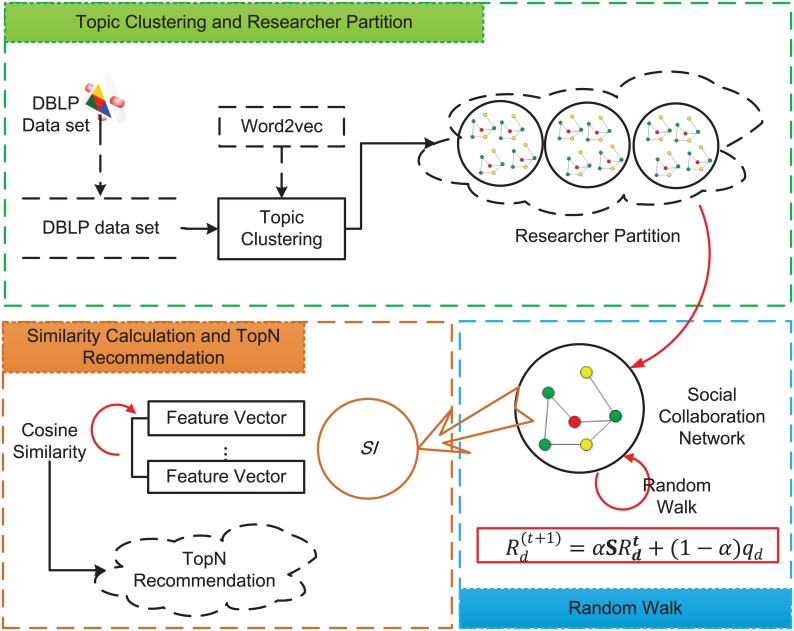
The architecture of CCRec. Depicts the three main components of CCRec: topic clustering and researcher partition, random walk, similarity calculation and top-N recommendation.

Our proposed recommendation scheme for CCRec is inspired by the reality and truth that a researcher usually desires to know other researchers who have similar research interests and strong influence in academia. As mentioned above, researchers often behave differently across multiple domains of interests. Such behaviors usually reveal the academic features of researchers in different domains. Besides, as a social-based model, Random Walk with Restart recommendation model (RWR) has been proved to be competent for calculating the rank score of nodes in social networks derived from co-authorship [5]. Researchers’ strength of influence in specific domains can be well reflected by RWR. In this work, we first adopt a content-based method to acquire multiple domains of interests. Secondly, we employ the social network-based method of RWR to measure the researchers’ strength of influence in different domains. In the final step of our design, we use the feature vector to evaluate the similarity of researchers and then obtain the recommendation list. The detailed process is described below and the corresponding pseudo-code is illustrated in Pseudo-code of the proposed scheme. Fig 1 depicts the three main components of CCRec.

### Topic Clustering and Researcher Partition

It is a content-based method for topic clustering and researcher partition, which generates various domains, and maps all researchers into these domains. In this section, we work on partitioning researchers according to academic topics. There are many topic modeling techniques involving Nature Language Processing (NLP), e.g. Word2vec [22], Latent Dirichlet Allocation (LDA) [23] and Probabilistic Latent Semantic Analysis (pLSA) [24]. which are well-developed and widely used and can solve our problem. However, We prefer word2vec here since that it is more suitable in this application scenario. LDA and pLSA generate the probability distribution of words and documents based on the co-occurrence of words and documents, which focus on describing their connotative topics. Word2vec runs based on contextual information (i.e. semantic, syntactic) of words. In the case of our CCRec model, the input data is a set of titles from all the papers created by each researcher. Different from long document, there are not rich topic information. However, the titles are complete sentences and full of contextual information. Hence, Word2vec can generate more accurate feature description for each researcher. For another reason, LDA-based models require prior distributions which are always difficult to define [25]. Word2vec provides an efficient implementation of the continuous of *bag-of-words* and *skip-gram* architectures for computing vector representations of words. It takes a text corpus as the input and produces the word vectors as the output. The final word vector file can be used as features in many NLP and machine learning applications. The word vectors can be also used for deriving word classes from huge data sets. This is achieved by performing K-means clustering on top of the word vectors. The output is a vocabulary file with words and their corresponding domain IDs. In our CCRec model, The input titles are split into many sequential words. In addition, it is necessary to filter out some “Stop words” (https://code.google.com/p/stop-words/), “of”, “the”, “and”, etc. When extracting words from titles, the set of preprocessed words can be used to outline the core contents of papers, which are signified as valuable and reliable corpus to denote a variety of academic topics. We run word2vec on this corpus, then the word vector can be automatically generated and the K-means clustering method is executed. The output file includes all academic words and their corresponding domain IDs. Now that the academic topics are clustered and the words are marked to each topic. We use a matrix **W** to denote these mapping relations. If a word *w* is marked to a topic *d*, *W*_*w*,*d*_ will be 1, otherwise 0.

**Algorithm 1** Pseudo-code of the proposed scheme

1: *SI* ← *init*()

2: **for** *d* in *D* **do** // Traverse domains set *D*.

3:  **S** ← *ComputeTransferMatrix*(*d*)

4:  *SI*_*d*_, *R*, *q* ← *InitVec*()

5:  **for** *k* ← 1 to *MaxIteration* **do**

6:   *diff* ← 0

7:   **for** *i* ← 1 to *len*(*q*) **do**

8:    *t* = *SI*_*d*,*i*_

9:    $SI_{d,i}=\alpha \sum_{j=1}^{len(q)}S_{i,j}*SI_{d,j}+\left(1-\alpha \right)q_{i}$

10:    *diff* ← *diff* + (*SI*_*d*,*i*_ − *t*)

11:   **end for**

12:   **if** *diff* < *MinDelta* **then**

13:    **break**

14:   **end if**

15:  **end for**

16: **end for**

17: **for** *u* *in* *U* **do**

18:  **for** *v* *in* *U* **do**

19:   *Sim*_*u*,*v*_ ← *CosSim*(*SI*_*u*_, *SI*_*v*_)

20:  **end for**

21: **end for**

22: RecommendTopN()

In addition, CCRec partitions researchers to specific domains through the following approaches: 1) Extract subject terms from the publication titles of researcher *u* (After filtering out the stop words). We use *T*_*u*_ to represent the subject terms set of researcher *u*. 2) Traverse all the subject terms in *T*_*u*_ and check the matrix **W**. The model tags the researcher for particular domains that contain these subject terms. We use the matrix **A** to denote the relations of a researcher and topics. Eq 1 shows the process of computing the matrix **A**.

$$A_{u,d}=\left\{\begin{array}{cc} 1 & \exists \;word\;w\in T_{u},\;that\;W_{w,d}=1\\ 0 & \forall \;word\;w\in T_{u},\;that\;W_{w,d}\neq 1 \end{array}\right.$$ (1)

**Fig 2:**
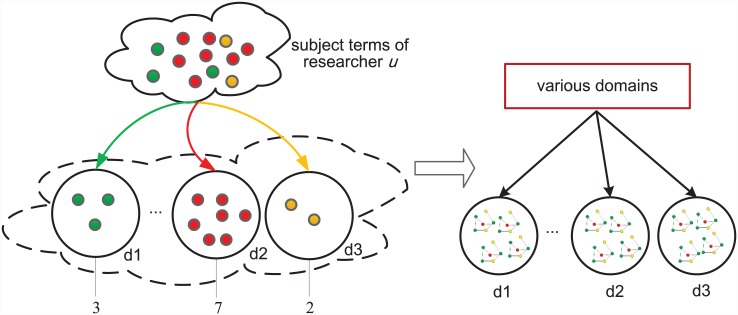
Researcher Partition. Illustrates an example of partition researchers to several domains.

According to the equation, if *A*_*u*,*d*_ = 1, the researcher *u* is marked to topic *d*. It should be emphasized that one researcher always belongs to several domains and there are also many researchers in one domain. Fig 2 illustrates an example. Assuming that CCRec extracts 12 subject terms from the publications titles of researcher *u*. After topic clustering, we can see that, three of these subject terms are affiliated to domain *d*_1_, seven to *d*_2_, and two to *d*_3_. Thus, researcher *u* is tagged for domains *d*_1_, *d*_2_ and *d*_3_. i.e. *A*_*u*,*d*_1__, *A*_*u*,*d*_2__, *A*_*u*,*d*_3__ are all equal to 1. Through this method, each domain contains numerous researchers.

### Feature Vector Calculation

As mentioned in Introduction, in general, researchers devote themselves to several adjacent domains. But in the case of attention and strength of influence in various domains, there are often some biases. To measure the influence of researchers, we define the Strength of Influence (*SI*) to denote the academic values (Rank Score) of researchers in different domains, which can be regarded as the feature vector elements of researchers. To compute *SI*, we adopt a social-network based model, the random walk with restart model RWR. In RWR, the rank score of a node is determined by the voting contributions from neighbors, which can be reflected by three factors: the out-degree of each neighbor, the count, and rank score of neighbors. The rank score in RWR can be used to represent the node’s importance in a social network. Therefore, we firstly model the social-networks. There are numerous researchers with similar research interests in domain *d*. Their co-author relationships are the most obvious and important links among researchers, which can be modeled by a social network *N*_*d*_. Thus, there are many co-author networks corresponding to different domains. We run the RWR model on the co-author networks *N*, the generated rank score of each node in the domain is used to denote academic values, i.e. *SI*. The core equation of the RWR model is shown in Eq (2) below:

$$R_{d}^{\left(t+1\right)}=\alpha SR_{d}^{\left(t\right)}+\left(1-\alpha \right)q_{d}$$ (2)

where *R*_*d*_ represents the rank score vector of all researchers in domain *d*, *q*_*d*_ is the initial vector *R*^0^, and *α* denotes the damping coefficient. In normal random walk model, *α* takes the value of 0.8, which has been confirmed in our experiments. **S** is the transfer matrix, which drives the random walker skipping to next node with a probability. RWR is an iterative process. After limited iterations, the vector *R*_*d*_ will be convergent. In this scenario, *SI*_*d*,*u*_ = *R*_*d*,*u*_. That is, the final value of the vector item *R*_*d*,*u*_ is the *SI* of researcher *u* in domain d.

In addition, with the help of RWR, the *SI* in various domains is quantified for each researcher. To measure researchers’ academic feature, we define the vector *F* with *SI* as *F*_*d*,*u*_ = *SI*_*d*,*u*_.

### Collaboration Recommendation Based on Feature Vector Similarity

CCRec recommends collaborators for researchers based on their similarities. To measure the academic feature similarities of researchers, we borrow a standard method, *cosine similarity (CS)*, as shown in Eq (3). CS is employed to define the similarity between two users *u*_1_ and *u*_2_ based on their feature vectors *F*_*u*_1__ and *F*_*u*_2__.

$$Sim\left(u_{1},u_{2}\right)=\frac{\sum_{i=1}^{n}\left(F_{u_{1},i}*F_{u_{2},i}\right)}{\sqrt{\sum_{i=1}^{n}F_{u_{1},i}^{2}}*\sqrt{\sum_{i=1}^{n}F_{u_{2},i}^{2}}}$$ (3)

Finally, we consider that researchers with high similarities have common interests. Therefore, they should be recommended to each other as potential academic collaborators. Hence, CCRec provides a *Top-N* recommendation list for each researcher.

## Results

We conducted various experiments on DBLP dataset [26] from year 2000 to 2014, a computer science bibliography website hosted at University of Trier, Germany, which indexes more than 2.3 million articles on computer science and contains many links to home pages of computer scientists. Each DBLP record contains these attributes: authors, title, page number, publishing year, crossref, proceedings or journals, etc. We modeled the co-author networks on DBLP by this principle: the co-author relation is created when two researchers co-authored at least one paper. Inevitably, there are some isolated authors who create their work independently. Thus, they nearly have no relationship with other scholars. Furthermore, we define these isolated authors as the weak nodes, since their degree values are 0. It is clear that the weak nodes have little impact on the random walk. Therefore, we ignore the weak nodes. We also ignore the problem of author disambiguation and assume that each researcher has an independent name. We extracted the subsets of the entire data using the required information, which are all in the field of data mining involving 34 journals and 49 conferences. If we model a large social network on the data, it contains 59659 nodes (authors), 90282 edges (co-author relations) and the average degree is 1.531. We extracted all titles of these publications and filter out “stop word”, the generated 104587 keywords can be the corpus of word2vec model. The detailed statistics are shown in Table 1. We divided the data set into two parts: the data before year 2011 as a training set, and the data after 2011 as a testing set.

**Table 1:** Statistics of Data Set from DBLP.

Nodes	Edges	Average Degree	words
59659	90282	1.513	104587

We embarked on benchmarking experiments involving CCRec. To evaluate the performance of CCRec model visually, we employed three popular metrics that are widely used in the recommender systems: *Precision*, *Recall* and *F1*[27]. The recommender system provides a recommendation list. Comparing with the collaborator list in testing data set, there are four sets. A: recommended and collaborated. B: recommended but not collaborated. C: not recommended but collaborated. D: not recommended and not collaborated. The performance of recommender system can be well reflected by the intersection ratio of the four sets.

The definition of precision is:

$$Precision=\frac{len(A)}{len(A)+len(B)}$$ (4)

Recall is:

$$Recall=\frac{len(A)}{len(A)+len(C)}$$ (5)

F1 is defined as:

$$F1=\frac{2(Precision*Recall)}{Precision+Recall}$$ (6)

**Fig 3:**
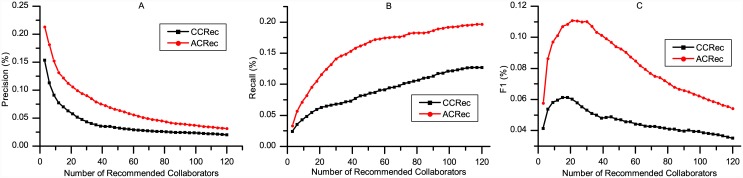
Performance of CCRec and ACRec on most valuable collaborators recommendation. The abscissas denote the length of recommendation list. The ordinates respectively represent the values of precision, recall and F1.

**Fig 4:**
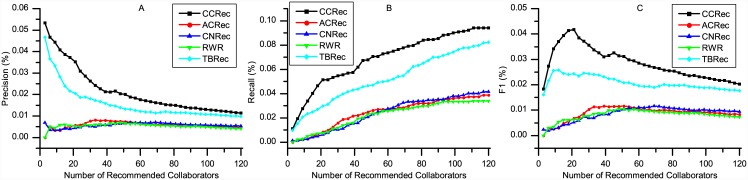
Performance of CCRec, ACRec, CNRec, TBRec and RWR on most potential collaborators recommendation. The abscissas denote the length of recommendation list. The ordinates represent the values of precision, recall and F1 respectively.

**Fig 5:**
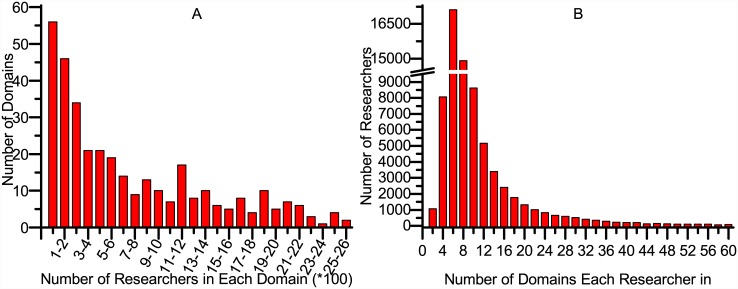
Statistics of data after topic clustering and researcher partition.

**Fig 6:**
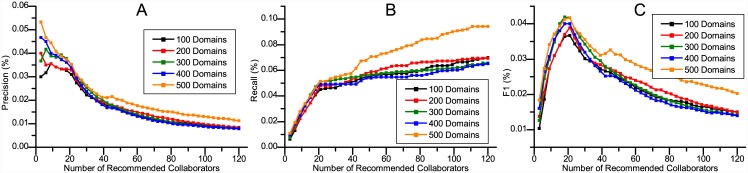
The impact of clustered domains number on CCRec. The abscissas denote the length of recommendation list. The ordinates represent the values of precision, recall and F1 respectively.

We compared CCRec with the following four approaches: a random walk based model (ACRec), a common neighbors based model (CNRec), a topic based model (TBRec) and the basic random walk model (RWR). ACRec: a random walk recommendation model based on collaboration networks [5], which uses three academic factors to recommend collaborators based on the RWR. ACRec was demonstrated to do excellently in MVC recommendation. CNRec: a common neighbors based recommendation model [2], which is famous and widely used in social-network based recommendation. Considering a researcher without any co-author relation to target researcher, the more common co-authors they have, the more probability it can be recommended. TBRec: a classical topic based model. In our experiments, we used LDA model to cluster the content of researcher’s publications and defined the feature vectors by the topic probability distribution. The recommendation is made by computing the cosine similarity of researcher’s feature vectors. RWR: conduct the recommendation by running a basic random walk with restart model on the whole co-author network [5]. ACRec, CNRec and TBRec have been proved to be effective in academic collaborators recommendation and can be delegated as the highly accurate recommender systems. RWR is conducted for baseline model. Three groups of experiments were conducted: 1) Find the MVCs, who may have known each other before, or be active in adjacent circles, 2) Recommend MPCs, who have never cooperated with the target researcher before, 3) Evaluate how domains clustering impact the performance of CCRec. For each experiment, there were 500 domains clustered. we randomly chose 100 constant researchers who are at least somewhat active in academic activities. We generated collaborators recommendation for these 100 researchers, and then computed the average of precision, recall and *F*1. Figs 3 to 6 show the experimental analysis and result. The source data of the figures is in S1 File.

All experiments were performed using a 64-bit Linux-based operation system, Ubuntu 12.04 with a 4-duo and 32 GHz Intel CPU, 4-G Bytes memory. All the programs were implemented in Python.

### Most Valuable Collaborators Recommendation

In our previous work [5], We proposed an ACRec model which generates the most valuable collaborators recommendation for researchers. In this section, we analyze the performance of CCRec and ACRec in terms of generating the most valuable collaborators recommendation. The comparative results are shown in Fig 3.

As shown in Fig 3, The number of recommended collaborators has an obvious influence on the metrics with a clear trend. In the case of CCRec, as shown in Fig 3(A), the precision drops when the number of recommended collaborators increases. At the same time, the recall in Fig 3(B) rises with the increase of recommendation list, which finally approximates to 20%. In the case of ACRec, it has the same trend with CCRec in terms of precision and recall. Thus it can be verified that precision and recall are a pair of contradictory metrics. In order to weigh the two metrics to maximize profit, Shani et al. [27] adopted the metric *F*1. Fig 3(C) describes the performance of CCRec and ACRec on *F*1. In case of CCRec model, *F*1 generally increases until the number of recommended collaborators is over 15, and then decreases gradually. Since point 15 is exactly the peak of *F*1, we can see that, CCRec performs best when recommending 15 collaborators to each researcher, and the *F*1 can reach 6.13%. However, in this scenario, ACRec gets its’ highest *F*1 score 11.01% at point 30.

A reflection of Fig 3 substantiates that ACRec outperforms CCRec in terms of generating the most valuable collaborators recommendation. This is because, ACRec is based on the link-importance guiding random walk, which considers the walk distance and rank score and seeks the most valuable collaborators who may have known each other before, or are active in adjacent circles. Thus, there is no obvious superiority for CCRec to find the most valuable collaborators in adjacent circles compared with ACRec. While, the superiority of CCRec lies in recommending MPCs as follows.

### Most Potential Collaborators Recommendation

We define the Most Potential Collaborators as collaborators who are worthy of being recommended and have never cooperated with the target researcher. Generating recommendations pertaining to the most potential collaborators is of great significance as the new collaborators are more meaningful and practical in the reality of academia. In this section, we explored the performance of CCRec, ACRec, CNRec, TBRec and RWR when recommending the most potential collaborators.

Fig 4 shows the performance of CCRec, ACRec, CNRec, TBRec and RWR in terms of precision, recall and *F*1 with the number of recommended collaborators increasing. It can be observed that CCRec significantly outperforms ACRec, CNRec, TBRec and RWR all the time on these three metrics. CCRec shows a downwards trend for precision and an upwards trend for recall rate. In the case of *F*1, it reaches a peak of 4.18% when recommending 21 researchers. From Fig 4, it is also evident that in relation to the generation of the most potential collaboration recommendations, CCRec and TBRec obviously outperforms ACRec, CNRec and RWR in terms of the evaluation metrics we utilized. Meanwhile, CCRec performs much better than TBRec.

Simply, CCRec outperforms other models with higher precision, recall and *F*1 on making the most potential collaborators. CCRec combines publications contents and collaboration networks to define the feature vectors which are used to represent each researcher. Such a procedure has distinct advantages (e.g. rich information, more accurate to represent researchers’ feature) in recommending new collaborators. Moreover, we can conclude that, our previous work ACRec is good at MVC recommendation, and CCRec performs better on MPC recommendation.

### Impact of Clustered Domains Number

For previous experiments in this work, we clustered 500 topics based on DBLP data set and matched researchers to different domains. Here we analyzed the statistics of these domains. As described in Fig 5, in terms of the number of researches in each domain, there are about 56 domains that contain up to 100 researchers, and two domains contain more than 2500 researchers. We can come to conclusion that, various domains show big difference in scales. What’s more, as shown in Fig 5(B), most researchers are active in 2 to 20 domains. However, there is no clear standard to make the domains division. The statistics show inconsistency with different clustering granularity. In this section, we exploit the impact of clustered domains number on the performance of CCRec.

We adopted the following experiment settings: (1) Evaluate how the precision, recall and *F*1 score change with the number of collaborators recommended, (2) Generate the most potential collaborators recommendation for those 100 researchers selected above and (3) Recommend 21 potential collaborators for each researcher. Fig 6 shows the experimental results.

According to Fig 6, the number of clustered domains do have certain effects on the performance of CCRec. If the number of clustered domains is appropriate, the *F*1 score achieves some enhancement. In this situation, when clustering the data mining academia into 500 domains, CCRec performs best over precision, recall and *F*1 score.

In summary, our proposed model, which combines content-based and social network-based methods shows improvement. Furthermore, in terms of precision, recall and *F*1, CCRec outperforms ACRec, CNRec, TBRec and RWR generating the most potential collaborators (MPCs) recommendations for academic researchers.

## Conclusions

In this paper, we focused on how to find researchers’ MPCs based on big scholarly data which is necessary in current academia. To this end, we proposed a novel recommendation model called CCRec, by combining the features of publications content and collaboration networks. A topic clustering model and a random walk model were adopted to obtain researchers features, and make MPCs recommendation for researchers. We conducted extensive experiments on a subset of DBLP data set to evaluate the performance of CCRec in comparison to other state-of-the-art models: ACRec, CNRec and TBRec, and a baseline model RWR. Our experimental results show that, CCRec outperforms other compared models in terms of precision, recall and *F*1 score. Due to the utilization of a topic clustering model, the problem of topic drift in academic research has been solved to some extent.

Our research on CCRec reveals that the combination of content-based and network-based methods can improve the generation of effective academic collaborations. Nonetheless, there is still room for future study in this direction. We extracted the titles of publications as the corpus of the topic clustering model, which are not more comprehensive than the abstract and main body of publications. Additionally, specific metrics should be utilized to evaluate the impact of topic drift. As future work, more experiments and studies will be conducted.

## Supporting Information

S1 File The source data of Figs 3–6. (XLSX)
